# Draft Genome Sequences of Vibrio atypicus Strains DSM 25292^T^ and TUMSAT1

**DOI:** 10.1128/MRA.01526-19

**Published:** 2020-02-06

**Authors:** Satoshi Kawato, Reiko Nozaki, Hidehiro Kondo, Ikuo Hirono

**Affiliations:** a Laboratory of Genome Science, Tokyo University of Marine Science and Technology, Tokyo, Japan; Indiana University, Bloomington

## Abstract

Vibrio atypicus is a Gram-negative bacterium associated with the digestive tract of penaeid shrimp. Here, we present the draft genome sequences of two V. atypicus strains, DSM 25292^T^ and TUMSAT1. Both assemblies were approximately 4.8 Mbp long, with GC contents of around 44%.

## ANNOUNCEMENT

Penaeid shrimp aquaculture has seen explosive growth in the past few decades, but the industry has been threatened by various infections (1). Vibriosis is one of the most serious bacterial diseases, with various causative species and virulence factors reported. Given the diversity of shrimp-pathogenic vibrios, it is important to catalog and deepen our knowledge regarding shrimp-associated bacteria.

During a survey of bacterial infections in cultured shrimp, we isolated *Vibrio* sp. strain TUMSAT1 from the hepatopancreas of a moribund kuruma shrimp (*Marsupenaeus japonicus*) that originated from a shrimp farm on Kumejima Island, Okinawa Prefecture, Japan, in May 2018. We conducted whole-genome sequencing of TUMSAT1 to find that the TUMSAT1 genome was substantially divergent from all other *Vibrio* genomes deposited in the NCBI database.

Meanwhile, 16S rRNA sequencing suggested that TUMSAT1 is closely related to Vibrio atypicus (2). However, V. atypicus genome assemblies were not available in public databases. Therefore, to resolve the relationship between TUMAST1 and V. atypicus, we sequenced strain DSM 25292^T^, the type strain of V. atypicus, which was originally isolated from the digestive tract of Chinese shrimp (Fenneropenaeus chinensis) (2).

Strain DSM 25292^T^ was purchased from the German Collection of Microorganisms and Cell Cultures GmbH. The two strains were cultured in heart infusion broth (Gibco) supplemented with 2.5% (wt/vol) NaCl, and genomic DNA was extracted using the standard cetyltrimethylammonium bromide extraction method. Paired-end libraries were prepared using the Nextera XT library preparation kit (Illumina, USA), following the manufacturer’s instructions, and sequenced using the MiSeq reagent kit, version 2 (300 cycles; Illumina). We processed the raw reads using fastp, version 0.20.0 (3), with default settings, and the resulting reads were *de novo* assembled using SPAdes, version 3.13.1 (4), with the following settings: careful, only-assembler, k = 21, 33, 55, 77, 89, 101, 113, and 125. Contigs shorter than 200 bp were discarded, and the remaining sequences were annotated using Prokka, version 1.13.3 (5, 6), with the following settings: force, rfam, kingdom *Bacteria*, Gram neg, genus *Vibrio*, usegenus. We calculated the DNA-DNA hybridization (DDH) between the two strains using the Genome-to-Genome Distance Calculator (GGDC), version 2.1 (http://ggdc.dsmz.de/ggdc.php) (7), and the DDH value calculated under formula 2 (sum of all identities found in high-scoring segment pairs [HSPs] divided by the overall HSP length) was adopted, as recommended by the program (7).

The draft genome assemblies of V. atypicus strain DSM 25292^T^ and TUMSAT1 resulted in 66 and 79 scaffolds, respectively, totaling 4,835,723 and 4,885,016 bp, with *N*_50_ values of 494,603 and 1,008,181 bp and GC contents of 43.90 and 43.74%, respectively. The numbers of predicted protein-coding genes were 4,347 for DSM 25292^T^ and 4,387 for TUMSAT1. The DDH between the two strains was 75.50%, suggesting that the two strains belong to the same species but may be delineated into two distinct subspecies.

### Data availability.

The V. atypicus draft genome assemblies are available in DDBJ/EMBL/GenBank with the accession numbers BLID01000001 to BLID01000066 (DSM 25292^T^), and BLIE01000001 to BLIE01000079 (TUMSAT1). The Illumina raw read data are available under the accession numbers DRR199590 (DSM 25292^T^) and DRR199589 (TUMSAT1).
